# Neuropsychiatric symptoms and gait/balance are predictors of functional trajectories in dementias

**DOI:** 10.1590/1980-5764-DN-2025-0455

**Published:** 2026-09-14

**Authors:** Rodrigo Gómez Rahmann, Michel Faggioni Piñones, Pedro Arellano Navarrete, Javiera Rodríguez Fuentes, Tahía Alarcón Sarria, Carolina Delgado Derio

**Affiliations:** 1 Centro de Apoyo Comunitario para Personas con Demencia Kintun, Santiago, Chile.; 2 Universidad de Santiago de Chile, Facultad de Ciencias Médicas, Santiago, Chile.; 3 Universidad de las Américas, Facultad de Salud y Ciencias Sociales, Santiago, Chile.; 4 Universidad del Desarrollo, Facultad de Medicina, Santiago, Chile.; 5 Universidad de Chile, Facultad de Medicina, Departamento de Neurología, Santiago, Chile.

**Keywords:** Neurocognitive Disorders, Activities of Daily Living, Behavioral Symptoms, Postural Balance, Walking Speed, Trastornos Neurocognitivos, Actividades Cotidianas, Síntomas Conductuales, Equilibrio Postural, Velocidad al Caminar

## Abstract

Dementias have heterogeneous functional decline trajectories, but little is known about the factors that shape this differential progression.

**Objective:**

To identify relevant predictors of functional decline in patients with dementia.

**Methods:**

A total of 76 subjects were included in this longitudinal retrospective, observational study. Functional outcome was assessed using the Technology-Activities of Daily Living Questionnaire (T-ADLQ), a 33-item scale encompassing basic, instrumental, and advanced activities of daily living (ADLs), with higher scores indicating greater deterioration. Participants were assessed three times: at baseline, and after a mean follow-up of 6.2 and 11.2 months, respectively. Cognitive and noncognitive variables were evaluated as predictors of functional decline using a linear mixed-effect model.

**Results:**

Females represented 63.2% of the sample. Subjects had a mean age of 79.5 (±7.9) years, 6.2 (±4.3) years of education, and a Global Deterioration Scale (GDS) score of 4.7 (±0.68) (median 5; range, 4-6). The linear mixed-effect model (R^2^=0.387) identified global cognition (Mini-Mental State Examination – MMSE), neuropsychiatric symptoms (NPI-Q), and gait/balance (Tinetti test and gait speed) as independent predictors of functional decline, with *𝛽* coefficients of -0.305, 0.299, and -0.281, respectively.

**Conclusion:**

This study underscores the importance of noncognitive predictors of functional trajectories in established dementia. In particular, neuropsychiatric symptoms and gait should be considered during the follow-up of people living with dementia to ensure tailored interventions and counseling for patients and their families. These findings support the inclusion of gait variables in further longitudinal studies on dementia prognosis.

## RESUMEN

Las demencias presentan trayectorias heterogéneas de deterioro funcional, pero se conoce poco acerca de los factores que determinan esta progresión diferencial.

**Objetivo:**

Identificar predictores relevantes del deterioro funcional en pacientes con demencia.

**Métodos:**

Se incluyeron 76 participantes en este estudio observacional, retrospectivo y longitudinal. El desenlace funcional se evaluó mediante el cuestionario de Tecnología y Actividades de la Vida Diaria (*Technology-Activities of Daily Living Questionnaire* – T-ADLQ), una escala de 33 ítems que abarca actividades básicas, instrumentales y avanzadas de la vida diaria (AVD), en la que puntuaciones más altas indican mayor deterioro. Los participantes fueron evaluados en tres momentos: al inicio y tras un seguimiento medio de 6,2 y 11,2 meses, respectivamente. Las variables cognitivas y no cognitivas se analizaron como predictores del deterioro funcional mediante un modelo lineal de efectos mixtos.

**Resultados:**

Las mujeres representaron el 63,2% de la muestra. Los participantes presentaron una edad media de 79,5 (±7,9) años, 6,2 (±4,3) años de escolaridad y una puntuación de 4,7 (±0,68) en la Escala de Deterioro Global (*Global Deterioration Scale* – GDS) (mediana, 5; rango, 4–6). El modelo lineal de efectos mixtos (R^2^=0,387) identificó la cognición global (*Mini-Mental State Examination* – MMSE), los síntomas neuropsiquiátricos (NPI-Q) y la marcha/equilibrio (prueba de Tinetti y velocidad de la marcha) como predictores independientes del deterioro funcional, con coeficientes β de -0,305, 0,299 y -0,281, respectivamente.

**Conclusión:**

Este estudio destaca la importancia de los predictores no cognitivos de las trayectorias funcionales en la demencia establecida. En particular, los síntomas neuropsiquiátricos y la marcha deben considerarse durante el seguimiento de las personas que viven con demencia para garantizar intervenciones y orientación adaptadas a los participantes y sus familias. Estos hallazgos respaldan la inclusión de variables relacionadas con la marcha en futuros estudios longitudinales sobre el pronóstico de la demencia.

## INTRODUCTION

With a global projection of 152,8 million cases by the year 2050, dementia incidence is rising, largely due to a growing population and increased life expectancy^1^. It is expected that, by 2050, the number of people with dementia in Latin American countries will reach 27 million^2^.

Functional decline, with a significant loss of independence in activities of daily living (ADL) caused by cognitive impairment, defines major neurocognitive disorder^3^, or dementia. The natural history of dementia consists of a progressive decline in functionality, communication, and independence^4^. In spite of this, functional and cognitive dementia trajectories are not homogeneous, and longitudinal studies have postulated subgroups with slower or faster progression^5^. However, little is known about the determinants of the trajectory of dementias after diagnosis^6^.

Traditionally, cognitive decline has been considered the leading driver of functional decline in dementia. However, it seems that it is not all about cognition, as neuropsychiatric^7^. comorbid conditions^8^, and motor capacities^9^ have been postulated as noncognitive dementia functional predictors.

Thereby, part of the heterogeneity in dementia progression could be attributed to these noncognitive determinants. Some of these variables could be suitable for compensation^5^, and, even if not, functional prognosis is important to adequately inform patients and families, as well as for the development of tailored healthcare^6^.

Our clinical research question was how relevant the cognitive and noncognitive variables obtained in our dementia healthcare practice could be in the functional prognosis of patients with dementia syndrome, including cognitive, neuropsychiatric, motor, biomedical comorbidity, and caregiver burden as potential predictors. We hypothesized the existence of noncognitive predictors of dementia functional trajectory.

## METHODS

### Study design

The study design was a retrospective observational primary analysis of a longitudinal database.

### Subjects

We included patients admitted to the Kintun program between January 2019 and June 2021.

Kintun is a dementia community center located in Santiago, Chile. The program consists of a multidisciplinary intensive intervention for people living with dementia and their families, encompassing medical, occupational, social worker, physiotherapy, and nutritional care, eventually adding a daycare center according to patient needs. Time to discharge is around 9 to 12 months.

Inclusion criteria were:

- a dementia syndrome of any cause;
- a minimal follow-up time of 6 months;
- functional evaluation at admission and at least 2 follow-up visits;
- all functional evaluations measured with the same scale.

Exclusion criteria were:

- very advanced dementia: a Global Deterioration Scale of Reisberg (GDS Reisberg) score of 7, as patients are unable to perform the motor component of the evaluations;
- lack of a reliable proxy.

Ethical approval for this study was obtained from the Mayor University Ethic Committee, Santiago, Chile, in accordance with the Declaration of Helsinki.

### Assessments

All patients underwent a comprehensive geriatric assessment every 3-6 months including demographic, cognitive, functional, motor, biomedical, and social evaluations, which are described below:

Demographic variables included age, biological sex, and years of education.

Clinical diagnoses were based on published diagnostic recommendations: National Institute on Aging Alzheimer’s Association workgroups on diagnostic guidelines for Alzheimer disease (AD)^10^, The Vascular Impairment of Cognition Classification Consensus Study^11^, Movement Disorders Society criteria for dementia associated with Parkinson Disease^12^, Dementia with Lewy Bodies Consortium criteria^13^, International Behavioural Variant FTD Criteria Consortium^14^, and the International Consensus Criteria for Primary Progressive Aphasia^15^. No biomarkers were available during the study period. The evaluation team rated the stage of dementia in the participants, according to GDS (GDS 4, mild dementia; GDS 5, moderate dementia; GDS 6, moderately severe dementia; and GDS 7, severe dementia)^16^, as suggested by the Chilean Dementia Plan guidelines^17^.

Cognitive performance and behavioral symptoms were evaluated using the Chilean version of the Mini-Mental State Examination (MMSE)^18^, and the Chilean version of the Neuropsychiatric Inventory Questionnaire (NPI-Q)^19^.

ADL were measured by the informant using the Technology-Activities of daily living questionnaire (T-ADLQ)^20^. The T-ADLQ is composed of 33 items that measure different ADLs. Each item is rated from 0 (no problem) to 3 (no longer capable of performing the activity). Higher percentage scores indicate greater deterioration, and the total score ranges from 0 to 100 points. A mild functional impairment ranges from 0 to 33, whereas 34 to 66 is considered moderate impairment. More than 67 points represents severe functional impairment.

T-ADLQ has the advantage of evaluating advanced, instrumental, as well as basic ADLs and has been validated in Chile^20^.

Gait/balance evaluation was performed through:

- gait speed, which was measured during normal walking using an 8-meter test, in which the time was recorded over the central 4 meters, allowing 2 meters for acceleration and 2 meters for deceleration. A normal gait speed was considered greater than 0.8 m.s^-121^ ; and
- the Tinetti test, which consists of two components (gait and balance).

This test allows prediction of the risk of falls. A total score of 18 or below is considered indicative of a high risk of falls, whereas a score between 19 and 23 indicates a moderate risk. Scores of 24 or higher indicate a low risk of falling^22^. Considering the high conceptual correspondence and predicted collinearity between gait speed and the Tinetti score, a composite score was created using the mean of both measures.

Handgrip strength was assessed with a digital dynamometer using the protocol with the participant seated in a chair with their arm at their side in the anatomical position. Normal ranges for the Chilean population were used, with cutoffs of ≤27 kg for men and ≤15 kg for women^23^.

The Charlson Comorbidity Index was calculated following the original methodology^24^.

Caregiver burden was assessed using the Chilean validated version of the abbreviated Zarit Burden Interview (ZBI-12)^25^.

Polypharmacy was defined as the presence of five or more prescriptions^26^, whereas the anticholinergic burden score was calculated based on the anticholinergic burden (ACB) scale and the German anticholinergic burden scale (GABS)^27^.

### Statistical analysis

Statistical analyses were performed using the jamovi project (2025, version 2.6) computer software. Participant baseline characteristics were summarized using means and standard deviations, medians and ranges, or frequencies and percentages, as appropriate. Outcome variable (T-ADLQ) follow-up was presented with means, standard deviations, medians, and ranges. Nonparametric Friedman’s repeated-measures ANOVA was performed to compare outcome means over time, and the chi-squared statistic was calculated to determine whether there was a statistically significant difference in the distributions of the repeated measures over time.

### Linear mixed-effect model

Repeated measures over time need to consider the evolution of each subject. Therefore, to capture individual trajectories, a linear mixed-effect model was used^28^, with each subject and their trajectory representing a “group,” the random component. In this study, this differential individual component is the random intercept; in other words, the different point at which each subject’s slope meets the y-axis. Based on the difference between the overall sample intercept and the random (individual) intercept, the interclass correlation coefficient (ICC) was calculated, representing the proportion of the variance explained by the individual trajectories of the subjects (groups), with values close to 1 indicating that this component is relevant and values close to 0 indicating that it is not necessary to consider this group component in the analysis.

In a linear mixed model, independent variables are considered the fixed component. Candidate fixed variables were analyzed after adjustment for age, sex, and education. Next, a multivariable linear mixed model was generated. In the multivariable mixed-effect model, the coefficient of determination is divided into conditional R^2^, which represents the sum of the random and fixed components, and marginal R^2^, which represents the proportion of the variance explained by the fixed components (the independent variables under study)^28^.

Fixed and outcome variables were standardized as Z scores to make them comparable by beta coefficients. A significance threshold of p<0.05 was adopted.

### Dementia phenotypes subgroup analysis

Given that certain dementia types (*i.e*., Lewy bodies dementias, which are associated with a high neuropsychiatric symptom burden), might disproportionately influence the statistical associations, an exploratory subgroup analysis was performed by separating patients with Alzheimer’s and non-Alzheimer’s phenotypes. Baseline characteristics were compared using the Mann-Whitney U test and Fisher’s exact test, as appropriate, and a linear mixed model was then performed for each group as described above.

## RESULTS

### Patients

**Figure 1:**
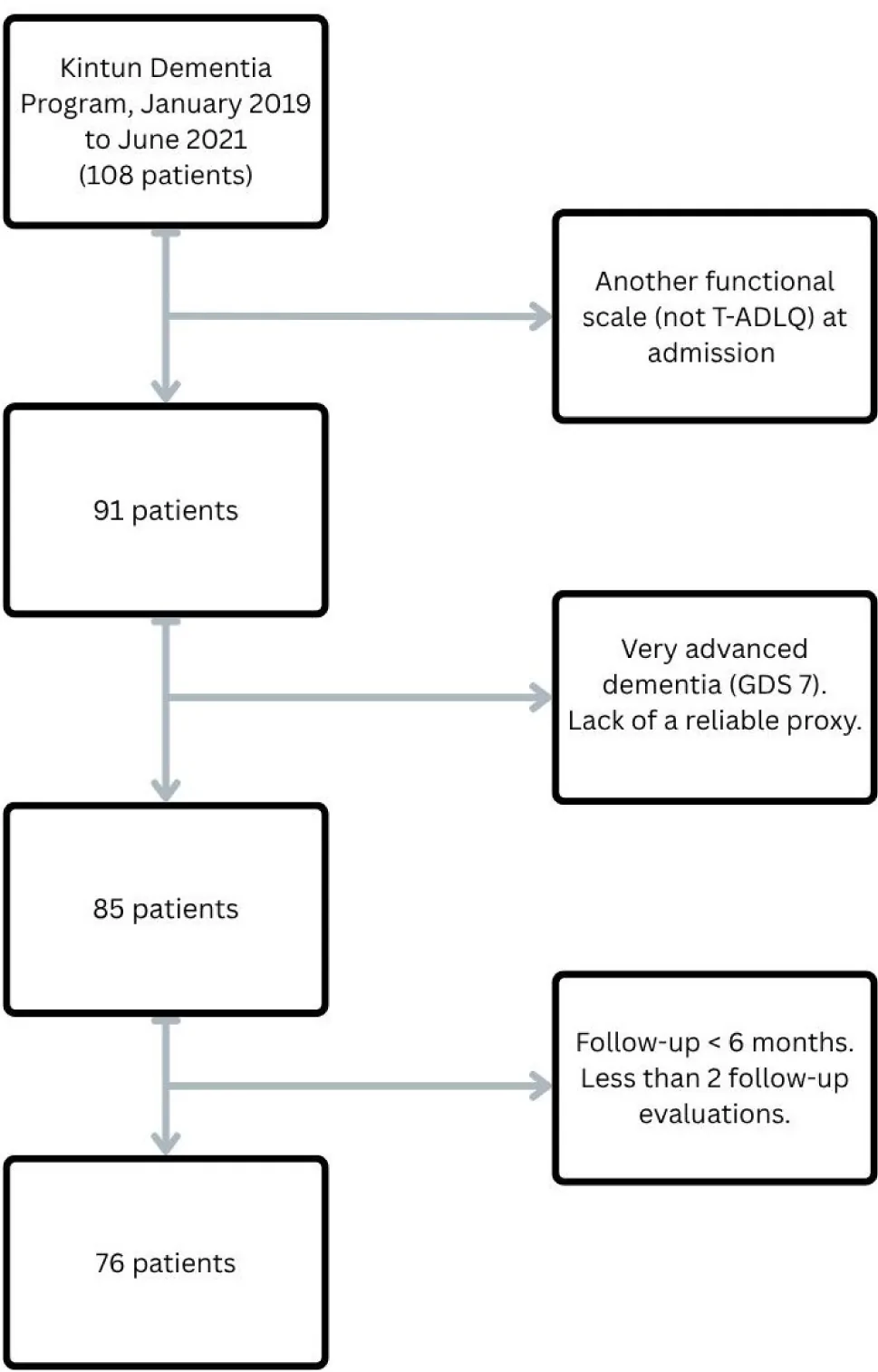
Patient flowchart.

Source: Created by the authors.

The initial sample comprised 108 subjects. After applying the inclusion and exclusion criteria, 76 patients were included in the study. Of note, T-ADLQ was progressively introduced at the Center during 2017; therefore, 17 patients were evaluated with another functional scale at admission and were not included (Figure 1).

### Demographics

A total of 63.2% of participants were women, with a mean age of 79.5 years and a mean of 6.2 years of education. Most patients (53.9%) had moderate dementia (GDS 5). The mean MMSE score was 15.4 (Table 1).

**Table 1:** Baseline characteristics of the study participants.

Baseline characteristics (n=76)					
		Mean/% (n)	SD	Median	Range
Sex (female)		63.2 (48)			
Age at admission (years)		79.5	7.9	79.5	53-96
Education (years)		6.2	4.3	6	0-17
T-ADLQ		58.3	18	61.3	15.4-94.7
GDS Reisberg		4.7	0.68	5	3.5-6
Dementia stage (by GDS)	Mild (GDS 4) Moderate (GDS 5) Moderately Severe (GDS 6)	31.6 (24) 53.9 (40) 14.5 (11)			
MMSE		15.4	5.3	16	4-30
NPI-Q		24.1	14	24.5	0-54
Zarit		24.5	18	21	0-94
Charlson comorbidity index		2	1.4	2	1-6
Anticholinergic burden		2.4	1.9	2	0-8
Polypharmacy		57.9 (44)			
Gait/balance	Gait speed (m.s^-1^)	0.64	0.22	0.66	0.140-1.15
	Tinetti test	19.6	4.4	20	10-28
Hand grip strength (kg)		18.2	7.2	17.2	6.2-39.6
Dementia phenotype	Alzheimer Disease Mixed (Alzheimer Disease+Vascular) Lewy Body Dementias Vascular Dementia Other causes (1 Frontotemporal dementia, 1 alcohol-related, 3 undefined)	46 (35) 32.9 (25) 9.2 (7) 5.3 (4) 6.6 (5)			

Abbreviations: SD, standard deviation; T-ADLQ, Technology-Activities of daily living questionnaire; GDS, Global Deterioration Scale; MMSE, Mini-Mental State Examination; NPI-Q, Neuropsychiatric Inventory Questionnaire.

### Dementia phenotypes

Dementia phenotypes comprised:

- AD (46%); and
- non-Alzheimer’s dementias: Mixed dementia (AD + vascular) (32.9%), Lewy Body dementias (LBD) (9.2%), vascular dementia (5.3%), and other causes (6.6%) (Table 1).

### Activities of daily living

The baseline outcome measure, T-ADLQ, was 58.3 (±18). Reassessment times were 6.2 (±1.7) and 11.2 (±2.6) months, with T-ADLQ scores of 64.0 (±17.3) and 68.7 (±16.3), respectively. Repeated-measures ANOVA (chi-square=51.611; gl 2; p<.001) demonstrated that T-ADLQ deteriorated significantly during the follow-up period.

### Determinants of functional decline

**Figure 2:**
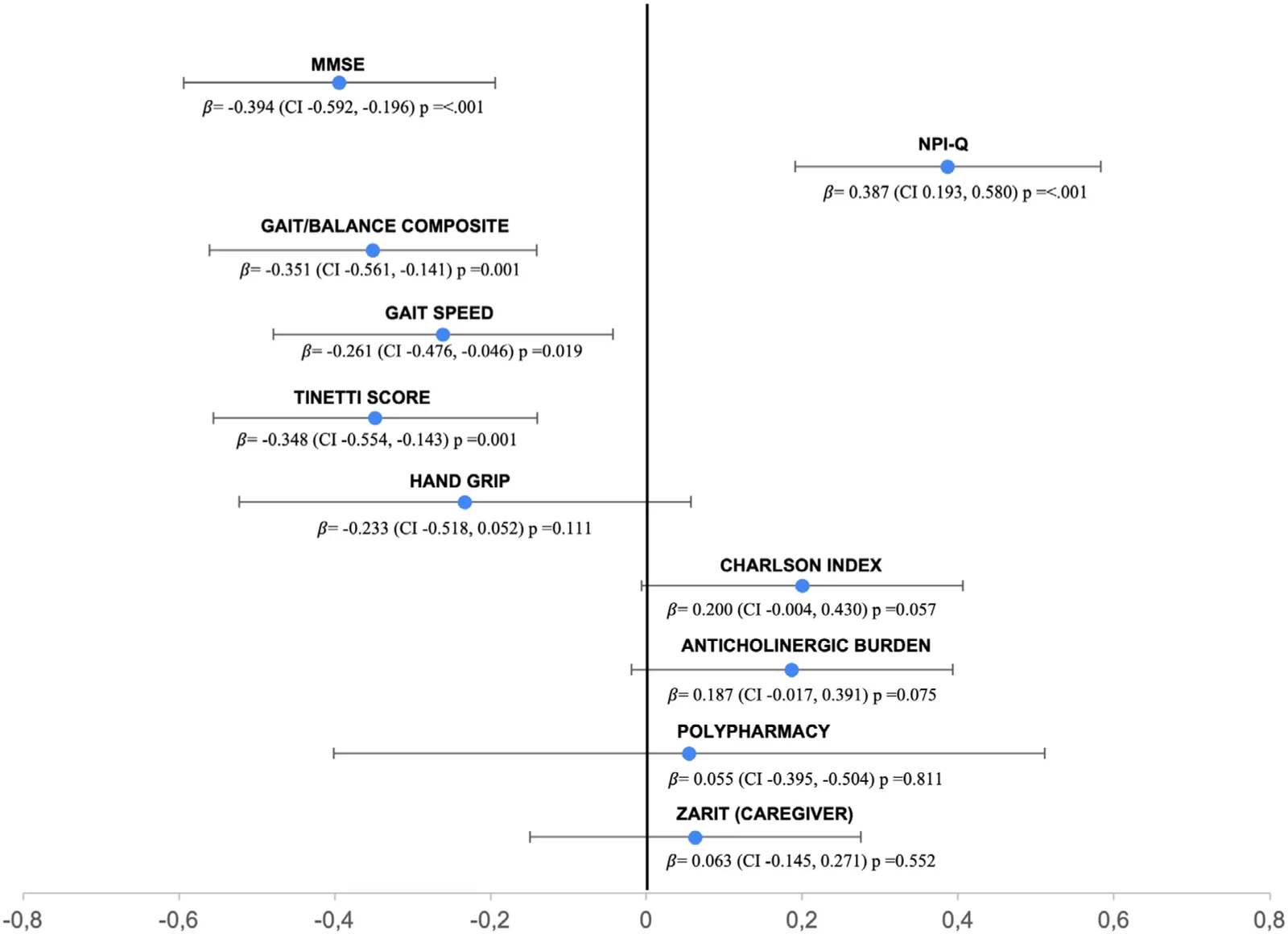
*𝛽* coefficients for fixed components (independent variables) in linear mixed effect model, adjusted by age, sex, and education.

Source: Created by the authors.

Figure 2 presents the initial analysis of candidate variables in the lineal mixed-effect model, adjusted for age, sex, and years of education. MMSE, NPI-Q, and the gait/balance composite (Tinetti and gait speed) were significant predictors of longitudinal functional impairment. On the other hand, neither handgrip strength, Charlson comorbidity index, anticholinergic burden, polypharmacy, nor caregiver burden reached statistical significance. Dementia phenotype was not tested because the non-Alzheimer dementia groups were too small.

The multivariate model (Table 2) resulted in a marginal R^2^=0.387. ICC was 0.728, indicating that grouping subjects in linear mixed-effect model was appropriate. The independent variables that remained significant and were therefore included in the final model, in descending order of effect size, were MMSE (*𝛽*=-0.305), NPI-Q (*𝛽*=0.299), and the gait/balance composite (*𝛽*=-0.281).

**Table 2:** Multivariable linear mixed-effect model.

Linear mixed-effects model for functional impairment trajectory Multivariate model Conditional R^2^=0.833; Marginal R^2^=0.387						
Standardized fixed coefficients (*𝛽* coefficients)						
Variables	Est(*𝛽*)	SE	95%CI		t	p-value
			Lower	Upper		
Intercept	0.009	0.086	-0.161	0.179	0.110	0.913
Time (1-0)	0.326	0.068	0.192	0.459	4,803	<.001
Time (2-0)	0.586	0.068	0.453	0.720	8,651	<.001
Age	0.136	0.094	-0.049	0.320	1,446	0.153
Sex (female vs.male)	-0.072	0.175	-0.416	0.272	-0.413	0.681
Education (years)	0.041	0.089	-0.134	0.215	0.458	0.648
MMSE	-0.305	0.090	-0.482	-0.128	-3,399	0.001
NPI-Q	0.299	0.088	0.126	0.473	3,399	0.001
Gait/balance composite	-0.281	0.090	-0.459	-0.103	-3,108	0.003
Gait speed	-0.263 *	0.091 *	-0.442 *	-0.083 *	-2,883*	0.005 *
Tinetti	-0.276 *	0.090 *	-0.455 *	-0.098 *	-3,053*	0.003 *
**Standardized Random Components**						
Groups	Name		Variance	SD	ICC	
Subject	(Intercept)		0.467	0.683	0.728	
Residual			0.174	0.418		

Abbreviations: 95%CI, confidence interval; SE, standard error; MMSE, Mini-Mental State Examination; NPI-Q, Neuropsychiatric Inventory Questionnaire; SD, standard deviation; ICC, Interclass Correlation Coefficient.

Notes: Number of Obs: 228, Number of groups: 76 subjects.

*Tinetti score and Gait speed were evaluated in separate models (not shown).

As expected, both components of the gait/balance composite, the Tinetti test and gait speed, showed moderate-to-high collinearity (rho=0.644). Alternative models revealed that both were significant predictors when tested individually (Table 2).

### Dementia phenotypes subgroup analysis

Table 3 details the characteristics of Alzheimer’s (n=35) and non-Alzheimer’s (n=41) phenotype groups. Both groups were comparable except for sex, for which a significant difference was found.

**Table 3:** Baseline characteristics of the phenotype subgroup analysis (Alzheimer’s vs. non-Alzheimer’s dementias).

Dementia Phenotype Subgroup Analysis			
Variables	Alzheimer’s dementia (n=35) Mean (SD)/% (n)	Non-Alzheimer’s dementias (n=41) Mean (SD)/% (n)	Mann-whitney u test / fisher’s exact test (p-value)
Age (years)	79.71 (9.50)	79.31 (6.26)	0.699
Sex (female)	82.85 (29)	46.34 (19)	0.002
Education (years)	6.28 (4.29)	6.04 (4.34)	0.971
Follow-up (months)	11.6 (2.49)	10.87 (2.79)	0.231
T-ADLQ (baseline)	55.21 (19.36)	60.98 (16.59)	0.179
GDS Reisberg	4.74 (0.70)	4.70 (0.64)	0.956
MMSE	14.68 (5.43)	15.87 (5.38)	0.446
NPI-Q	22.02 (14.14)	25.87 (13.86)	0.217
Gait Speed (m/s)	0.67 (0.23)	0.61 (0.20)	0.205
Tinetti test	20.54 (4.56)	18.75 (4.10)	0.068

Abbreviations: SD, standard deviation; T-ADLQ, Technology-Activities of daily living questionnaire; GDS, Global Deterioration Scale; MMSE, Mini-Mental State Examination; NPI-Q, Neuropsychiatric Questionnaire.

**Figure 3:**
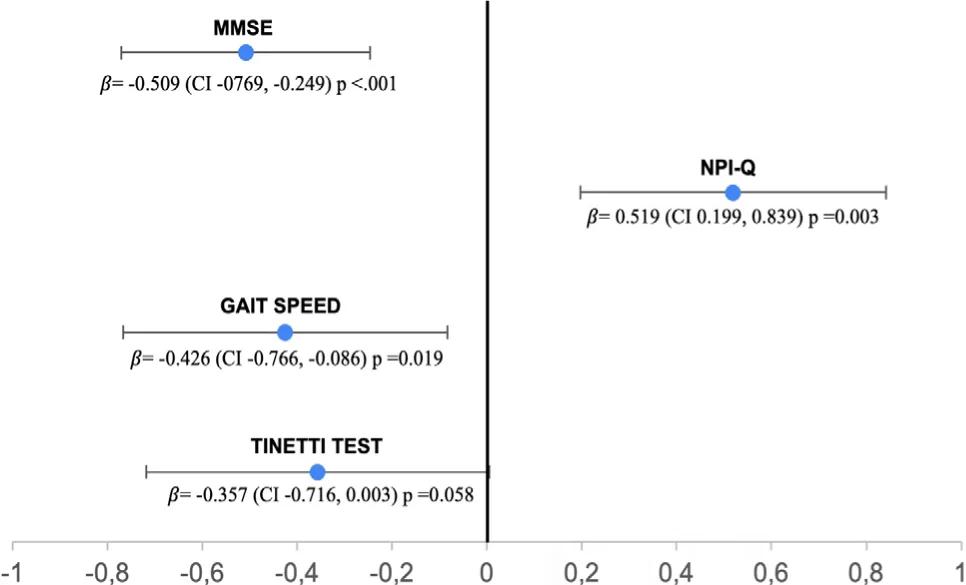
Subgroup analysis of Alzheimer’s Disease phenotype patients (n=35). *𝛽* coefficients for fixed components (independent variables) in linear mixed effect model, adjusted by age and sex.

Source: Created by the authors.

**Figure 4:**
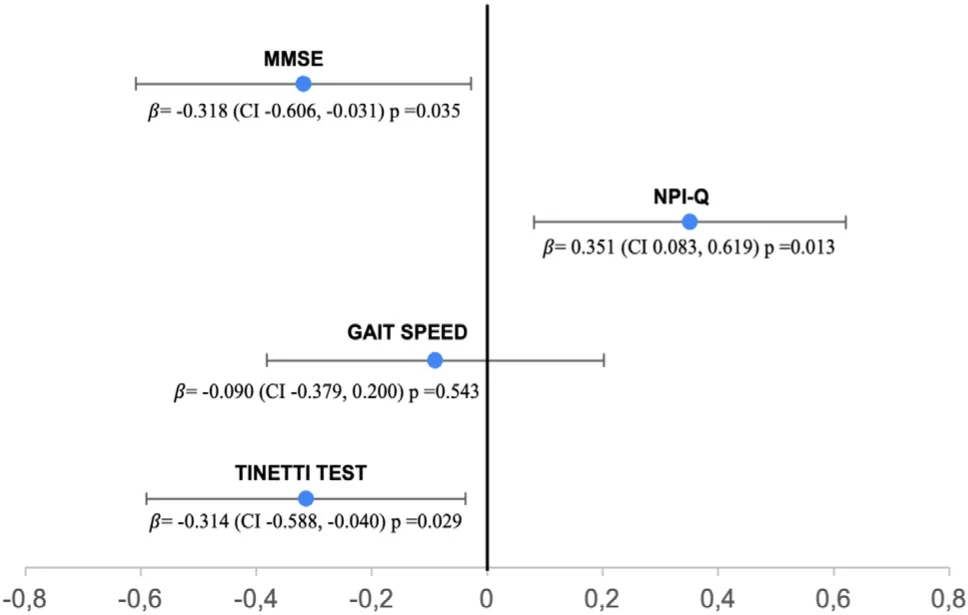
Subgroup analysis of Non-Alzheimer phenotype dementia patients (n=41). *𝛽* coefficients for fixed components (independent variables) in linear mixed effect model, adjusted by age and sex.

Source: Created by the authors.

Exploratory dementia phenotype analysis using linear mixed-effect models (adjusted for age and sex) showed that MMSE and NPI-Q were significant functional predictors in both subgroups (Figures 3 and 4). Gait speed (but not the Tinetti test) was a functional predictor in the Alzheimer’s group (Figure 3), whereas the Tinetti test (but not gait speed) was a functional predictor in the non-Alzheimer’s group (Figure 4).

## DISCUSSION

This longitudinal, retrospective, observational study identified cognitive and noncognitive independent predictors of dementia functional trajectory: global cognitive performance, behavioral symptoms, and gait/balance performance.

Neuropsychiatric symptoms (NPS) have been previously evaluated in longitudinal studies. Edwin et al.^29^, in a 3-year follow-up of 442 patients with Alzheimer’s and non-Alzheimer’s dementias, found that a high NPI-Q score was associated with more rapid functional progression^29^. In contrast, Haaksma et al. did not find a significant relationship between NPI-Q and dementia progression groups in a prediction model of latent classes including 1,120 AD cases; however, they used the Katz ADL scale, which only covers basic ADLs^5^. Meanwhile, in a 5-year follow-up of 196 patients with AD and LBD, Borda et al. found that NPI-Q was an independent predictor of both basic and instrumental ADLs^30^. Our findings are in line with these studies, and the subgroup analysis seems to support that neuropsychiatric symptoms are as relevant functional predictors in Alzheimer’s as in non-Alzheimer’s dementias^31,32^.

NPS could be related to accelerated functional impairment through various proposed mechanisms: an increased risk of falls, hospitalization, decompensation of chronic illnesses, isolation, diminished use of sensory compensatory strategies, and, possibly, involvement of common brain areas or networks implicated in functional and behavioral regulation^30^. Polypharmacy, anticholinergic burden, and increased caregiver stress could represent additional mechanisms; however, in the present study, none of these variables was significantly related to functional progression.

Taken together, these findings reinforce that neuropsychiatric symptoms should be systematically included in dementia assessment and follow-up. However, it remains to be demonstrated whether intensive management of NPS improves functional outcomes in patients with dementia^29,30^.

Regarding gait and balance evaluation, these measures have been notably absent from longitudinal studies evaluating established dementia progression^5,6,29,31,32,33^. Nevertheless, a few cross-sectional studies have assessed this issue, with mixed results. On the one hand, Clemmensen et al., studying the timed up and go test and the 30-second sit-to-stand test in 185 patients with AD, did not find significant correlations between physical performance and ADLs^34^. On the other hand, other two cross-sectional studies did find significant correlations between physical performance and functionality. Yu et al.^35^, evaluating 28 participants, reported that the Short Physical Performance Battery (SPPB) was associated with ADLs^35^, and, more recently, Tsiakiri et al.^36^ found that the Tinetti score was a predictor of instrumental ADLs in 76 patients with dementia^36^.

Gait speed has been consistently related with progression during predementia stages, in what has been termed motoric cognitive risk syndrome^37,38^. Recently, Collyer et al.^39^, in a 6-year follow-up of 19,114 participants, found that dual decliners (*i.e.,* in both, cognition and gait speed) had the highest risk of incident dementia^39^. Based on the available evidence, gait speed has been incorporated into recommendations on early noncognitive markers of dementia^40^. Our findings suggest that, even in established dementia stages, gait speed remains a predictor of functional trajectory, configuring a continuum ranging from predementia to mild and moderate dementia stages.

The Tinetti test, on the other hand, has not been specifically evaluated in relation to progression during predementia stages. However, the occurrence of multiple falls has been proposed as a marker of future cognitive decline and the emergence of motoric cognitive risk syndrome in nondemented individuals^41^.

From a pathophysiological standpoint, proposed mechanisms relating gait to cognitive progression include shared cardiovascular risk factors and common neural networks affected by microvascular disease or Alzheimer’s pathology^39^. Recently, dual decline was associated with a broader pattern of atrophy (temporal, parietotemporal, and perisylvian regions) and greater microstructural deterioration in several white matter tracts than isolated memory decline or isolated gait decline in ageing participants^42^.

On the other hand, it cannot be ruled out that, in the moderate-to-advanced dementia group in the present sample, parkinsonism or gait apraxia secondary to frontal or subcortical histopathological progression may have contributed to both gait/balance and functional impairment^43^.

The subgroup analysis suggests a possible differential association of gait/balance with functional progression between the Alzheimer’s phenotype (gait speed) and the non-Alzheimer’s phenotype (Tinetti test). Gait speed appears to be a sensitive functional predictor during Alzheimer’s predementia stages, according to the aforementioned motoric cognitive risk syndrome^37,38,39,40^, which is consistent with the present findings. Regarding the absence of an association between fall risk, balance, and the Alzheimer’s subgroup, the Tinetti test has not been directly evaluated; however, previous studies in nondemented older adults have considered falls a risk factor for motoric cognitive risk syndrome, but not a direct risk factor for incident dementia^41,44^. It may be hypothesized that, in established Alzheimer’s-type dementia, gait speed remains a stronger functional predictor than fall risk. Conversely, the relevance of the Tinetti test in the non-Alzheimer’s phenotype could be related to the broader motor (*e.g.*, bradykinesia, paresis) and cognitive (executive and visuospatial) impairments characteristic of dementias with a prominent motor component, such as Lewy body and vascular dementias^45^. For the same reasons, it seems counterintuitive that gait speed was not a functional predictor in non-Alzheimer’s dementias in the present study. Nevertheless, Anang et al. found that longitudinal dementia risk in Parkinson disease was predicted by falls, freezing of gait, and clinical gait evaluation, but not by the timed up and go test^46^, which broadly aligns with the present findings, in which balance and qualitative gait assessed by the Tinetti test appeared more relevant to the progression of non-Alzheimer’s dementias than gait speed. It may be speculated that, in dementias with a prominent motor component, gait speed impairment is already integrated into the clinical trajectory at the time of dementia diagnosis. Therefore, gait speed may no longer predict functional impairment, whereas balance and fall risk may be more informative in this context.

The present findings warrant the inclusion of physical variables in future prospective, larger longitudinal studies of dementia trajectory. From a clinical perspective, the results support the systematic assessment of gait characteristics in patients with dementia to ensure comprehensive clinical assessment, counseling, and care. Moreover, gait speed and the Tinetti test are simple, inexpensive, and easily applicable in primary healthcare settings. Finally, there is growing evidence supporting physical activity within the context of multicomponent preventive interventions^47^, as well as its therapeutic effects in established Alzheimer’s dementia^48^.

The main strengths of this study were its longitudinal design, the broad range of variables evaluated, and the close follow-up. Among the limitations are the small sample size, retrospective methodology, convenience sample from a single center, and limited follow-up duration. Moreover, the study may have been underpowered to detect significant effects of handgrip strength, the Charlson comorbidity index, and anticholinergic burden. On the other hand, further studies may prefer latent class prediction models to account for the longitudinal behavior of all variables rather than only the outcome^6^. Finally, the influence of the intensive multidisciplinary intervention on the evolution of some of the studied variables cannot be excluded.

In conclusion, the present study underscores the importance of a multidimensional approach to people with dementia and their families, systematically integrating physical and behavioral assessments into dementia counseling, care, and research.

## Data Availability

The datasets generated and/or analyzed during the current study are available from the corresponding author upon reasonable request.
